# Molecular basis for the thermostability of Newcastle disease virus

**DOI:** 10.1038/srep22492

**Published:** 2016-03-03

**Authors:** Guoyuan Wen, Xiao Hu, Kang Zhao, Hongling Wang, Zhenyu Zhang, Tengfei Zhang, Jinlong Yang, Qingping Luo, Rongrong Zhang, Zishu Pan, Huabin Shao, Qingzhong Yu

**Affiliations:** 1 Institute of Animal Husbandry and Veterinary Sciences, Hubei Academy of Agricultural Sciences, Wuhan 430070, China; 2 US National Poultry Research Center, Agricultural Research Services, United States Department of Agriculture, Southeast Poultry Research Laboratory, Athens, GA 30605, USA; 3 State Key Laboratory of Virology, College of Life Sciences, Wuhan University, Wuhan 430072, China; 4 Hubei Key Laboratory of Animal Embryo and Molecular Breeding, Wuhan 430070, China

## Abstract

Thermostable Newcastle disease virus (NDV) vaccines have been used widely to protect village chickens against Newcastle disease, due to their decreased dependence on cold chain for transport and storage. However, the genetic basis underlying the NDV thermostability is poorly understood. In this study, we generated chimeric viruses by exchanging viral genes between the thermostable TS09-C strain and thermolabile LaSota strain using reverse genetics technology. Evaluations of these chimeric NDVs demonstrated that the thermostability of NDV was dependent on the origin of HN protein. Chimeras bearing the HN protein derived from thermostable virus exhibited a thermostable phenotype, and vice versa. Both hemagglutinin and neuraminidase activities of viruses bearing the TS09-C HN protein were more thermostable than those containing LaSota HN protein. Furthermore, the newly developed thermostable virus rLS-T-HN, encoding the TS09-C HN protein in LaSota backbone, induced significantly higher antibody response than the TS09-C virus, and conferred complete protection against virulent NDV challenge. Taken together, the data suggest that the HN protein of NDV is a crucial determinant of thermostability, and the HN gene from a thermostable NDV could be engineered into a thermolabile NDV vaccine strain for developing novel thermostable NDV vaccine.

Newcastle disease (ND) is a highly contagious and often fatal avian disease, and poses considerable threat to the poultry industry worldwide^1^. The causative agent of ND is virulent strains of Newcastle disease virus (NDV), which is an enveloped virus with a non-segmented, single-stranded RNA genome of negative polarity, and belongs to the genus *Avaluvirus* within the family *Paramyxoviridae*^2^. The 15.2-kb RNA genome contains six genes encoding the nucleoprotein (NP), phosphoprotein (P), matrix (M), fusion (F), hemagglutinin-neuraminidase (HN), and large polymerase (L) proteins. The open reading frame of each gene is flanked by short 5′ and 3′ untranslated regions (UTRs), which contain conserved gene start (GS) and gene end (GE) sequences, respectively. Two additional proteins, V and W are produced by RNA editing during the transcription of P gene^3^. NDV proteins can perform several functions *in vitro*, including the hemagglutination (HA) ─ aggregation of erythrocytes, neuraminidase activity (NA) ─ removal of neuraminic acid from molecules containing carbohydrate, and hemolysis ─ lysis of erythrocytes by fusion with the cell membrane. HA and NA activity are functions solely of the surface protein HN^4^,^5^. Another surface protein F, together with the HN protein, is required for hemolysis and infectivity^6^,^7^. These functions, which are part of the process of infection of host cells, have been previously studied^8^,^9^.

The effective prevention and control of avian infectious diseases usually depend on vaccination in China and many other countries. However, most live vaccines are sensitive to heat, and subsequently require a cold chain to maintain the quality of vaccines during transport and storage. It is expensive to keep vaccines at low temperature and the cold chain may consume up to ~80% of the total cost of vaccination programs^10^. Moreover, the cold chain is not always reliable. Temperature excursions outside the optimal temperature range are frequently observed during transport and storage^11^,^12^, due to inappropriate cold chain equipment, human error, and power shortages^13^,^14^,^15^. The situation is even worse in developing and less-developed countries. The failure of cold chain may lead to the rapid loss of potency and inadequate protection against disease. It is estimated that roughly 50% of vaccine products discarded because of the poor thermostability^16^. Therefore, the development of thermostable vaccines that could be partially or completely independent of a cold chain is of great importance.

Most of the NDV vaccine strains are thermolabile, such as LaSota and B1, and a few of them are thermostable, such as V4 and I_2_^17^,^18^. The thermostable V4 strain was isolated from the proventriculus of healthy fowl in Australia in 1966^19^. The I_2_ strain was selected among 45 Australia isolates by evaluating their immunogenicity in chickens, ability of spread among host, and thermostability after heating at 56 °C for 30 min^18^. The viral mean titer of freeze-dried V4 vaccine reduced from 10^10.4^ to 10^9.3^ EID_50_ per vial after incubation at 27–32 °C for 14 days^20^. The V4 vaccine coated onto the carrier feed products was stable for a minimum of 3 weeks at 21–27 °C^21^. When diluted with 1% gelatine, the I_2_ vaccine could still produce an antibody response after storage at 22 °C for 12 weeks^18^. The thermostable vaccines have been used widely to protect village chickens against ND, especially in the developing and less-developed countries^22^,^23^,^24^. However, the mechanism of thermostability of these successful vaccine strains is poorly understood.

To discover the molecular basis of the NDV thermostable phenotype, several thermostable NDV strains were sequenced. By comparison with the thermolabile strains, Yusoff *et al.* found an Arg (403) deletion in the HN protein of the thermostable V4-UPM strain, suggesting this deletion might be responsible for the thermostablility of NDV^25^,^26^. However, Kattenbelt *et al.* did not find the Arg (403) deletion in the HN protein of other thermostable strains, and proposed that the amino acid differences in the L protein might be responsible for the NDV thermostability^27^. We previously sequenced the complete genome of thermostable strain TS09-C, derived from V4, and did not find any conserved sequence alternations between TS09-C and thermolabile strains^28^. Therefore, it seems impossible to predict the possible thermostable determinants of NDV by sequence alignment.

Development of infectious cDNA clones (ICs) of NDV has enabled genetic approaches for identifying the role of a viral protein, protein domains and UTRs in viral replication and virulence^29^,^30^,^31^, and for developing vaccine vector for other pathogens^32^,^33^,^34^. Previously, we have successfully constructed the ICs of NDV thermostable strain TS09-C and thermolabile strain LaSota^35^,^36^, which allowed us to investigate the genetic traits that are responsible for the thermostability of NDV.

In this study, we generated chimeric Newcastle disease viruses by exchanging viral genes between the thermostable TS09-C strain and thermolabile LaSota strain using reverse genetic technology. Thermostability of these chimeric viruses was examined to identify the thermostable determinants of NDV. Results demonstrated that the HN protein is a crucial determinant of NDV thermostability. Furthermore, the newly generated chimeric virus containing the TS09-C HN gene in the backbone of LaSota strain increased the thermostability and conferred a complete protection of chickens against lethal NDV challenge.

## Results

### Thermostable determinant of NDV is located within the region spanning from F to HN gene

**Figure 1:**
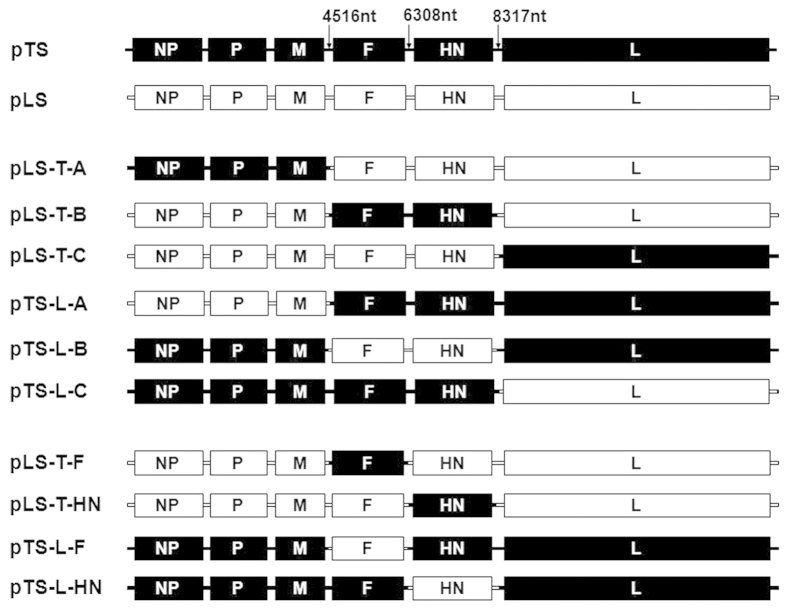
Schematic representation showing constructions of chimeric NDVs. Dark and white bars represent the genes of TS09-C and LaSota strain, respectively. Corresponding nucleotide numbers where the gene fragments were fused by using In-fusion cloning technology were depicted.

To identify the NDV thermostable determinant, the genome of NDV was divided into 3 genomic fragments, named A, B, and C (Fragment A contains NP, P, and M genes; B contains F and HN genes; C contains L gene). Six chimeric ICs of NDV were constructed by the exchange of genomic fragments (A, B, or C) between the ICs of TS09-C and LaSota strain (Fig. 1). Chimeras rLS-T-A, rLS-T-B, rLS-T-C, rTS-L-A, rTS-L-B, and rTS-L-C were rescued from their respective chimeric ICs, and examined for thermostability (Table 1). As shown in Table 1, among three chimeras on the background of thermostable TS09-C strain, only rTS-L-B showed significantly decreased thermostability (P = 0.017 versus rTS09-C), and changed from thermostable phenotype into thermolabile one. Among the chimeric viruses on the background of the thermolabile LaSota strain, only rLS-T-B increased thermostability (P = 0.0032 versus rLaSota) and became the thermostable phenotypic virus. The genomic fragment B contains both the F and HN genes. Therefore, the thermostable determinant of NDV should be located within the regions spanning from F to HN gene.

### NDV HN protein is the crucial thermostable determinant

**Figure 2:**
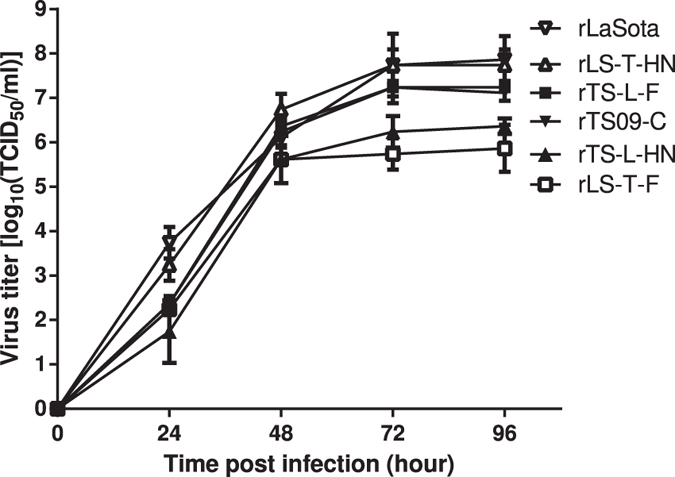
Growth kinetics of NDV rTS09-C, rLaSota, and chimeric viruses rLS-T-F, rLS-T-HN, rTS-L-F, and rTS-L-HN in BHK-21 cells. BHK-21 cells were infected (MOI = 0.1) with different chimeric viruses. At the indicated time points, media from infected cells were collected and titrated for virus yield in BHK-21 cells. Means and standard deviations were shown from three independent experiments (n = 3).

To narrow down the region where the thermostable determinant resides, four additional chimeric ICs were constructed in which the F or HN gene was exchanged between the ICs of the TS09-C and LaSota strains (Fig. 1). Chimeras, rLS-T-F, rLS-T-HN, rTS-L-F, and rTS-L-HN were rescued and evaluated for biological properties relative to rTS09-C and rLaSota strains. rLS-T-F and rTS-L-HN displayed a slower growth dynamics with 2.22 and 0.96 log_10_ lower than their parental viruses in BHK-21 cells, respectively (P < 0.0015) (Fig. 2 and Table 2). However, there were no significant differences in growth titers among these four chimeras in embryonated eggs (P > 0.10) (Table 2). All chimeras retained the lentogenic pathotype with ICPI being 0.00 and MDT >110 h. It is interesting to note that the rLS-T-F chimera had a slightly decreased virulence with a MDT >168 h, compared with its parental rLaSota; whereas rTS-L-F had a slightly increased virulence with MDT being 113 h, compared with its parental rTS09-C (Table 2).

**Figure 3:**
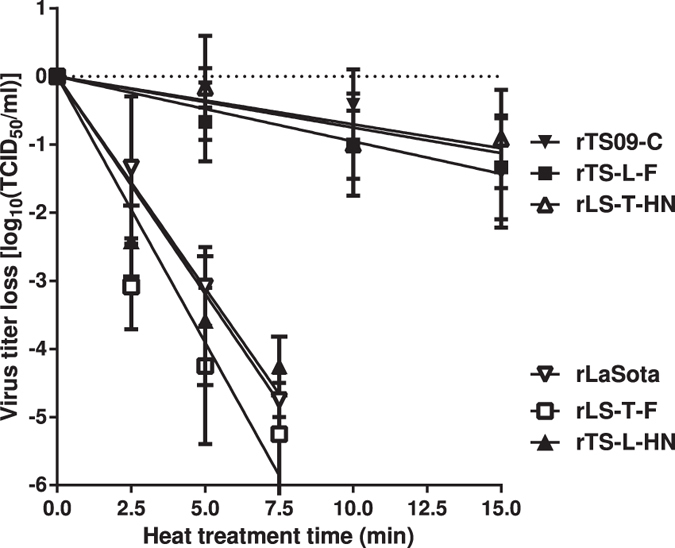
*In vitro* infectious thermostability test of NDV rTS09-C, rLaSota, and chimeric viruses rLS-T-F, rLS-T-HN, rTS-L-F, and rTS-L-HN. Heat-inactivation kinetics of infectivity of the different NDVs were determined at 56 °C. The titer loss of infectivity was represented on a logarithmic scale as a function of heat treatment time. Values were the averages of three independent tests (Mean ± SD, n = 3).

As shown in Fig. 3, the mean times for 90% decrease in infectivity of rLS-T-F, rLS-T-HN, rTS-L-F, and rTS-L-HN were 1.3, 14.0, 10.5, and 1.6 min, respectively. The infectivity inactivation rates of rLS-T-HN and rTS-L-F were ≥6-fold slower than rLS-T-F and rTS-L-HN, similar to that of rTS09-C strain. According to the criteria for the thermostability of NDV strains^17^, chimeric viruses containing HN gene of TS09-C strain, such as rLS-T-HN and rTS-L-F, belong to thermostable viruses, whereas those containing HN gene of LaSota strain, such as rLS-T-F and rTS-L-HN, belong to thermolabile viruses. These data demonstrate that the crucial determinant of thermostability of NDV is located within the HN protein.

### HA and NA activities of TS09-C HN protein are more thermostable than those of LaSota

**Figure 4:**
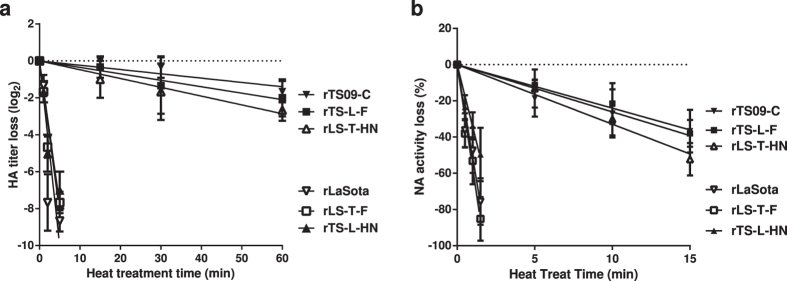
*In vitro* HA and NA thermostability test of NDV rTS09-C, rLaSota, and chimeric viruses rLS-T-F, rLS-T-HN, rTS-L-F, and rTS-L-HN. Heat-inactivation kinetics of (**a**) HA activity and (**b**) NA activity of the indicated NDV strains were determined at 56 °C. The remaining fractions of (**a**) HA activity and (**b**) NA activity were represented on a logarithmic and percent scale, respectively, as a function of heat treatment time. Values were averages of three independent experiments (Mean ± SD, n = 3).

The HN of NDV is a multi-functional protein and possesses HA and NA activities. To investigate the role of HA and NA activities of HN protein in NDV thermostability, three thermostable rNDVs (rTS09-C, rTS-L-F, and rLS-T-HN) and three thermolabile viruses (rLaSota, rTS-L-HN, and rLS-T-F) were examined *in vitro* by performing the HA and NA thermostability assays. Figure 4a showed that the mean times for 90% HA activity decrease of the thermostable rNDVs containing the HN gene of TS09-C were ≥69.2 min, while those of thermlabile rNDVs encoding the LaSota HN gene were ≤2.1 min. The HA inactivation rates of rNDVs containing the TS09-C HN gene were at least 32-fold slower than those of viruses containing the LaSota HN gene at 56 °C. Similar results were obtained from the NA thermostable assay, the NA thermostability of rNDVs containing the TS09-C HN gene were much higher than those of viruses containing the LaSota HN gene (Fig. 4b). These results demonstrated that both HA and NA activities of TS09-C HN protein were more thermostable than those of LaSota strain.

### Thermostable chimeric virus rLS-T-HN protects chickens against virulent NDV challenge

To evaluate the protection conferred by the thermostable chimeric virus rLS-T-HN against NDV challenge, four groups of birds were immunized with the rLS-T-HN, rTS09-C, rLaSota, or PBS and challenged with a lethal dose of NDV strain F48E9 at 14 dpv. As shown in Table 3, all birds immunized with the rLS-T-HN, rTS09-C, or rLaSota survived from the virulent NDV challenge without showing any signs of ND, whereas the chickens in the PBS group displayed conjunctivitis and severe depression from 2 to 4 days post-challenge (dpc) and mortality of 100% at 5 dpc. The average NDV HI titers were log_2_ 6.50, 2.78, and 7.33 for rLS-T-HN, rTS09-C, and rLaSota vaccinated group, respectively (Table 3). The rLS-T-HN induced a significantly higher HI titer than rTS09-C strain (P = 6.4E-5) and a comparable HI titer to the parental rLaSota strain. The newly developed NDV strain rLS-T-HN not only retained the thermostability of the HN gene from the donor virus, but also retained the immunogenicity of its parental backbone vaccine strain LaSota, while conferring 100% protection from clinical disease and survival of chickens challenged with virulent NDV.

### The rLS-T-HN vaccine induces antibody response after storage at 30 °C for 16 days

To estimate the potency loss of vaccine during the storage, the rLS-T-HN, rTS09-C, and rLaSota liquid vaccine were stored at a relatively high room temperature (30 °C) for periods of 1 to 16 days, and their subsequent infectivities were titrated in embryonated eggs. As shown in Table 4, the virus titers of rLS-T-HN, rTS09-C, and rLaSota vaccines were 6.31, 6.65, and 1.91 log_10_ EID_50_/ml, respectively, after 16 days of storage. When these three vaccines were used to vaccinate chickens after the 16-day storage, the rLS-T-HN and rTS09-C vaccine induced NDV antibody response, the average HI titers were log_2_ 6.37 and 2.89, respectively. However, there was no antibody response in any chickens to the rLaSota vaccine. These data demonstrated that the rLS-T-HN liquid vaccine can maintain its vaccine potency for at least 16 days of storage at 30 °C, but the commonly used commercial LaSota vaccine will not retain its potency at the same condition of storage.

## Discussion

In the present study, we have shown that the NDV HN protein markedly affects the viral thermostability *in vitro*. The replacement of HN gene in the thermolabile strain LaSota with the HN gene from the thermostable strain TS09-C resulted in significantly increased thermostability. In contrast, the replacement of HN gene in TS09-C strain with that of LaSota strain led to the decreased thermostability. It is notable that no other NDV genome region was able to alter the viral phenotype of thermostability, indicating the significance of NDV HN protein in thermostability. Moreover, the newly developed thermostable chimeric virus rLS-T-HN was able to induce a higher level of antibody response than TS09-C strain and conferred a complete protection of chickens against the lethal NDV challenge.

Since the first thermostable NDV V4 strain was isolated in 1966 in Australia, some lentogenic thermostable NDV strains have been discovered and several of them have been used as vaccines to protect village chickens against ND in developing and less-developed countries^18^. However, the genetic basis and molecular mechanism underlying the NDV thermostability are largely unknown, mainly due to the lack of a molecular tool such as an infectious clone of thermostable NDV. Previous sequence comparisons among NDV strains have revealed some hints of the molecular basis for the thermostability. Tan *et al.*^25^ and Yusoff *et al.*^26^ compared the HN sequences of NDV strain V4 and V4-UPM with higher thermostability than the original V4, and suggested the Arg (403) deletion in HN gene might attribute to the thermostability. However, Kattenbelt *et al.*^27^ did not find such a deletion in several heat-tolerant NDV isolates, including the V4-UPM. Instead, they found that the majority of sequence differences between the I_2_ parental stock and themostable I_2_ master seed virus were located in L protein, proposing the alternations in L protein were responsible for the thermostable phenotype^27^. Here, by utilizing the recently developed IC of thermostable NDV TS09-C strain, derived from the V4 strain, we identified that the NDV thermostable determinant resided in the HN, but not in the L or other proteins. Although it is well-known that the HN is responsible for the infection and the pathogenesis of NDV^37^,^38^, the role of HN in viral thermostability has not been proved until this study.

The NDV HN is a surface glycoprotein with multi-functions, such as receptor recognition in the host cells, receptor removal, and interaction with F protein to promote fusion. Our results presented in this study have shown that the HN protein has one more biological function being responsible for thermostability of NDV, but the precise mechanism of the HN protein mediating NDV thermostability is still unclear. It was proposed that binding of HN with the sialic acid receptor of the host cells triggers the interaction of HN with F, leading to the conformational change of F to initiate fusion^39^,^40^. The first step of NDV infection was the binding of HN with cell receptor by utilizing the HA activity of HN protein. When the LaSota virion was exposed to environmental heat, the HA activity of virion was quickly inactivated, and the subsequent binding of cell receptor did not occur and the viral infection could not be initiated. However, when the TS09-C virion was subjected to heat, all the viral activities could be retained, such as HA, fusion, and polymerase activity. This allowed the initiation of infection and a complete cycle of viral replication. Therefore, we speculated that the HN protein affects viral thermostability through regulating the HA thermostability and subsequent initial infection of NDV. This is in agreement with the finding that no single thermostable NDV strain was found to have the character of thermolabile HA activity (I^+^Ha^−^)^17^. In contrast to this situation, there were several thermolabile NDV strains with thermotable HA activity (I^−^Ha^+^)^17^. The possible explanation was that although the HA activity was retained after heat-treatment of the I^−^Ha^+^ viruses, the other viral activity, such as fusion, or polymerase activity may be inactivated quickly, and subsequent viral replication cycle could not be completed.

Evaluation of the pathogenicity of these rNDVs has shown an interesting aspect that the rNDVs bearing the F gene of TS09-C (the rTS09-C, rTS-L-HN, and rLS-T-F viruses) were markedly more avirulent than those containing the LaSota F gene (the rLaSota, rLS-T-HN, and rTS-L-F viruses). The F cleavage site of TS09-C is ^112^G-K-Q-R-R-L^117^, with an isolated basic amino acid and a paired contiguous amino acids^28^. While that of LaSota is ^112^G-R-Q-G-R-L^117^, with two isolated basic amino acids. The F cleavage site is a major determinant of NDV virulence^30^,^41^, so this is possibly due to the different F cleavage site between the TS09-C and LaSota strain.

Thermostable vaccines with the reduced independence on a cold chain can provide benefits by decreasing transport and storage costs, ensuring potency, and reducing waste, which are greatly helpful to extend the global coverage of vaccines^42^,^43^. However, most NDV vaccines are sensitive to heat. It is of great importance to improve the thermostability of vaccines. Wang *et al.*^44^ integrated the biomimetic nucleating peptides onto the capsid of enterovirus type 71 (EV71) vaccine strain by reverse genetics, so that a mineral exterior on the virus was spontaneously formed under physiological conditions. After which, the self-biomineralized EV71 vaccine exhibited greatly improved thermostability. In NDV, the LaSota strain is one of the most popular vaccine strains used worldwide. Here, we significantly improved the thermostability of LaSota strain by replacing the corresponding regions in LaSota strain with the HN gene of TS09-C strain. The newly developed chimeric virus rLS-T-HN showed approximately 9-fold improved thermostability at 56 °C, and retained similar immunogenicity to the parental LaSota strain. The rLS-T-HN liquid vaccine could induce a high level of antibody response in chicken after storage for 16 days at 30 °C. Although both rLS-T-HN and rTS09-C have the same HN gene, which is mainly responsible for induction of HI titer, the difference in HI titers between rLS-T-HN and rTS09-C was noticed. The results suggested that in addition to HN gene, other NDV genes, such as F or L gene, could also affect the immunogenicity/HI titer of NDV^45^,^46^. It would be of special interest to replace the HN gene in other NDV or paramyxovirus vaccine strains with that of TS09-C strain, and observe the effects of the HN gene replacement on viral thermostability and immunogenicity.

In conclusion, the major NDV thermostable determinant associated with the HN protein has been identified. By utilizing the approach of replacing the HN gene in LaSota strain with that of TS09-C strain, a new thermostable NDV was generated and proved to protect host from lethal NDV challenge. The improved understanding of the genetic basis of NDV thermostability will promote rational design of live thermostable NDV or other paramyxovirus vaccine strains with less dependence on cold chain, especially for the developing and less-developed countries, to reduce the vaccination costs and improve the vaccine efficacy.

## Methods

### Animals and ethic statement

Embryonated eggs from SPF Leghorn chickens were purchased from Merial-Vital, Beijing, China. SPF chickens were hatched in a contained environment, and raised in negative pressure isolators for animal work. This study protocol was approved by the Institutional Animal Care and Use Committee of the Hubei Academy of Agricultural Sciences, and was carried out in accordance with the approved guidelines.

### Cells and Viruses

The BHK-21 [C-13] cells (ATCC^®^ CCL-10^TM^) were maintained in Dulbecco’s modified Eagle medium (DMEM; Life Technologies) and were incubated at 37 °C in 5% CO_2_. The highly virulent NDV strain F48E9 was received originally from China Veterinary Culture Collection Center, and was propagated in 10-day-old SPF embryonated chicken eggs and stored at −70 °C before use in a challenge study.

### Construction of chimeric ICs

The ICs of NDV thermostable strain TS09-C and thermolabile strain LaSota, named pTS and pLS, respectively, were constructed previously^35^,^36^. cDNAs amplified from TS09-C or LaSota strain by reverse-transcriptase polymerase chain reaction (RT-PCR) were cloned into modified pBR322 vector to obtain pTS or pLS, respectively. The sequences of all primers used in the construction of full-length cDNA clones are available upon request.

Chimeric ICs pTS-L-A, pTS-L-B, pTS-L-C, pTS-L-F, and pTS-L-HN were constructed by replacing the genomic fragments 1-4516nt, 4517-8317nt, 8318-15186nt, 4517-6308nt, and 6309-8317nt, respectively, in pTS with the counterpart fragments from the LaSota strain (Fig. 1). For example, to construct the plasmid pTS-L-A, the pTS fragment was PCR amplified as a vector, by using the *PFUUltra* II Fusion HS DNA polymerase (Agilent) and vector-specific primers to exclude the genomic fragment 1-4516nt of TS09-C strain. The genomic fragment 1-4516nt of LaSota strain was amplified using gene-specific primers with the same polymerase and the pLS plasmid. Subsequently, the pTS-L-A was generated by the ligation of two PCR products, the genomic fragment 1-4516nt of LaSota strain and the pTS vector fragment, using an In-Fusion PCR clone kit (Clontech). Similarly, chimeric ICs pLS-T-A, pLS-T-B, pLS-T-C, pLS-T-F, and pLS-T-HN were constructed by replacing the genomic fragments 1-4516nt, 4517-8317nt, 8318-15186nt, 4517-6308nt, and 6309-8317nt, respectively, in pLS, with the counterpart fragments from pTS (Fig. 1).

### Rescue and propagation of chimeras

Rescue of the chimeric viruses was performed by co-transfecting the chimeric ICs, and NP, P and L supporting plasmids into MAV-T7-infected BHK-21 cells using Lipofectamine 2000 (Life Technologies), as described previously^36^. The rescued viruses were amplified by inoculating 100 μl of the cell lysate into the allantoic cavity of 10-day-old SPF chicken embryos and incubating the embryos at 37 °C. After 4 days of inoculation, the allantoic fluids were harvested and the rescued viruses were examined by HA assay using 0.5% chicken red blood cells^47^.

### Virus titration and growth kinetics

The titers of NDV chimera stocks were determined by performing the HA assay, the 50% tissue culture infectious dose (TCID_50_) assay on BHK-21 cells in the presence of 0.2 μg/ml TPCK-trypsin, and the 50% egg infectious dose (EID_50_) assay in 10-day-old SPF embryonated chicken eggs^47^. To determine the growth kinetics, monolayers of BHK-21 cells were infected with NDV chimeras at 0.1 MOI for 1.5 h, then washed three times with Phosphate-buffered saline (PBS), and overlaid with medium containing 2% fetal bovine serum (FBS). The media from infected cells were harvested at the indicated time points, and the titrations of virus from media were performed using the TCID_50_ assay.

### Pathogenicity tests

The pathogenicity of the NDV chimeras was determined by conducting the mean death time (MDT) assay in 10-day-old SPF embryonated chicken embryos and intracerebral pathogenicity index (ICPI) assay in 1-day-old SPF chickens^47^.

### Thermostability test

Aliquots of 1.0 ml undiluted allantoic fluids infected with NDV chimeras were sealed in air-tight vials. The vials were submerged into a water bath at 56 °C, and were transferred to an ice-cold water bath to stop the thermal inactivation at the indicated time points. The infectivity and HA activity of these heat treated viruses in vials were titrated by performing the TCID_50_ assay in BHK-21 cells and the standard HA assay^47^, respectively. The decreased infectivity and HA activity of these viruses were shown on a logarithmic scale as the heating time increase. Regression lines were plotted from 4 time points. The different time points were used for the heat-treatment test of the thermostable and thermolabile viruses. For infectivity titer, the time points of thermostable virus were 0, 5, 10, and 15 min, while those of thermolabile virus were 0, 2.5, 5, and 7.5 min; For HA activity, the time points of thermostable virus were 0, 15, 30, and 60 min, while those of thermolabile virus were 0, 1, 2, and 5 min. The thermostability of NDV was shown as the mean time for l log_10_ and 3.32 log_2_ decrease (90% decrease) in infectivity and HA activity, respectively. The survival percentage of viral infectivity was determined by dividing the infectivity after heating for 5 minutes by the infectivity before heating.

### Virus NA assays

The NA activity was determined by the fluorescence-based assay using a Neuraminidase Assay Kit (Beyotime, Jiangsu, China) according to the manufacture’s instruction. In brief, 10 μl of allantoic fluid infected with NDV chimeras was added to 70 μl of detection buffer, followed by adding 10 μl of NA fluorogenic substrate and 10 μl of water. After incubation at 37 °C for 30 min, the cleavage of NA fluorogenic substrate by NDV chimeras produces fluorescence with an excitation wavelength of 322 nm and an emission wavelength of 450 nm, measured using a Fluorescence Spectrophotometer (Hitachi, F-7000). NA activity was shown as the fluorescence intensity of samples above the background values of non-infected allantoic fluid.

### Immunization and challenge experiments

To evaluate the protective efficacy conferred by NDV chimera against lethal NDV challenge, forty 1-day-old SPF chickens were randomly divided into 4 groups of 10 birds. Chickens in group 1, 2, and 3 were inoculated with 0.1 ml of rLS-T-HN, rLaSota, and rTS09-C, respectively, at 10^6.0^ EID_50_/ml via the intranasal and intraocular (IN/IO) routes. Birds in group 4 were inoculated with 0.1 ml of PBS, served as unvaccinated control. At 14 days post-vaccination (dpv), all birds were challenged with virulent NDV strain F48E9 at a dose of 10^4.0^ EID_50_ per bird via the IN/IO routes. Blood samples were collected from each bird prior to the challenge, and detected for NDV-specific antibody by hemagglutination inhibition (HI) assay^47^. The NDV strain LaSota was used as the antigen. Birds challenged with NDV strain F48E9 were monitored daily for clinical signs and mortality for 2 weeks.

### Preservation of liquid vaccine

Aliquots of undiluted allantoic fluids infected with the NDV rLS-T-HN, rTS09-C, or rLaSota were incubated at 30 °C, and titrated in embryonated eggs at 0, 4, 8, 12, and 16 days post incubation. After preservation at 30 °C for 16 days, chickens were inoculated with 0.1 ml of the undiluted vaccines as described in the immunization experiment without challenge. The NDV HI titers were determined at 14 dpv^47^.

### Statistical analysis

Statistical differences between the chimeric viruses and their corresponding backbone viruses (rTS09-C, or rLaSota) were measured by one-way analysis of variance (AVONA) at a 5% level of significance with the use of GraphPad Prism 5.0 (GraphPad Software, California San Diego, USA).

## Additional Information

**How to cite this article**: Wen, G. *et al.* Molecular basis for the thermostability of Newcastle disease virus. *Sci. Rep.* **6**, 22492; doi: 10.1038/srep22492 (2016).

## Figures and Tables

**Table 1:** Thermostable characteristics of NDV rTS09-C, rLaSota, and chimeric viruses at 56 °C*.

Virus	Time for 90% decrease in activity (min)		Percent Survival§ (%)	Thermostable Phenotype	Virus Titer (log_10_EID_50_/ml)
	Infectivity	HA activity			
rTS09-C	13.3 ± 4.1^a^	142.6 ± 38.7	42 ± 12	I^ + ^Ha^+^	9.50 ± 0.43
rTS-L-A	9.2 ± 2.4^a^	95.1 ± 24.4	28 ± 8	I^ + ^Ha^+^	9.32 ± 0.25
rTS-L-B	1.7 ± 0.3^b^	2.0 ± 0.6	0.085 ± 0.024	I^−^ Ha^−^	8.95 ± 0.38
rTS-L-C	10.1 ± 1.9^a^	140.4 ± 24.9	32 ± 11	I^ + ^Ha^+^	9.53 ± 0.14
rLaSota	1.6 ± 0.2^c^	1.7 ± 0.5	0.078 ± 0.025	I^−^ Ha^−^	9.25 ± 0.25
rLS-T-A	1.7 ± 0.1^c^	2.4 ± 0.3	0.085 ± 0.032	I^−^ Ha^−^	9.41 ± 0.14
rLS-T-B	9.5 ± 3.1^d^	91.4 ± 31.6	30 ± 9	I^ + ^Ha^+^	9.04 ± 0.50
rLS-T-C	1.9 ± 0.5^c^	2.3 ± 0.3	0.27 ± 0.04	I^−^ Ha^−^	9.33 ± 0.13

^*^Data shown represented the averages of three independent experiments (Mean ± SD, n = 3). The different lowercase letters in the same backbone virus group indicate statistically significant differences (*P* < *0.05*) of the time for 90% decrease in infectivity.

^§^Of infectivity after heating for 5 minutes.

**Table 2:** Pathogenicity and growth titer of NDV rTS09-C, rLaSota, and chimeric viruses rLS-T-F, rLS-T-HN, rTS-L-F, and rTS-L-HN.

Virus	MDT (h)	ICPI	HA titer* (log_2_)	Virus titer*	
				Allantoic fluid (log_10_EID_50_/ml)	BHK-21 cells (log_10_TCID_50_/ml)
rTS09-C	>168	0.00	8.67 ± 1.15	9.50 ± 0.50^a^	7.08 ± 0.34^c^
rTS-L-F	113	0.00	9.67 ± 1.15	9.06 ± 0.52^a^	7.07 ± 0.60^c^
rTS-L-HN	>168	0.00	10.33 ± 0.58	9.38 ± 0.29^a^	6.12 ± 0.44^d^
rLaSota	118	0.00	11.33 ± 0.58	9.25 ± 0.58^b^	8.21 ± 0.68^e^
rLS-T-F	>168	0.00	8.67 ± 1.15	8.97 ± 0.66^b^	5.99 ± 0.25^f^
rLS-T-HN	121	0.00	9.17 ± 0.76	9.17 ± 0.14^b^	7.94 ± 0.29^e^

^*^The titers were expressed as the average titers of virus from three independent tests (Mean ± SD, n = 3). The different lowercase letters in the same backbone virus group indicate statistically significant differences (*P*  <  *0.05*) of the virus titers.

**Table 3:** Immunogenicity and protective efficacy of NDV chimeric virus in 1-day-old SPF chickens*.

Virus Immunized	No. of birds	HI titer (log_2_)	Protection rate (%)
rLS-T-HN	10	6.50 ± 0.58^a^	10/10 (100)
rTS09-C	10	2.78 ± 0.56^b^	10/10 (100)
rLaSota	10	7.33 ± 0.52^a^	10/10 (100)
PBS	10	0.00^c^	0/10 (0)

^*^All birds in each group were immunized with a dose of 10^5.0^ EID_50_ in a 0.1 ml volume via IN/IO routes and challenged with 10^4.0^ EID_50_ NDV strain F48E9 at 14 days post-vaccination. The HI titers were expressed as log_2_ mean ± SD. The different lowercase letters indicate statistically significant differences (*P* < *0.05*) of the HI titers.

**Table 4:** Survival of NDV chimeric virus as liquid vaccine at 30 °C.

Virus	Titers* after incubation for days				
	0	4	8	12	16
rLS-T-HN	9.26 ± 0.25	8.67 ± 1.04	8.08 ± 0.72	7.24 ± 0.25	6.31 ± 0.43§
rTS09-C	9.45 ± 0.42	9.16 ± 0.34	8.49 ± 0.43	7.90 ± 1.53	6.65 ± 0.87§
rLaSota	9.38 ± 0.33	7.32 ± 0.14	5.66 ± 0.82	2.57 ± 1.13	1.91 ± 0.78‡

^*^50% egg infectious dose, log_10_ per 1 ml. The mean titers were shown from three independent tests (Mean ± SD, n = 3).

^§^SPF chickens vaccinated by IN/IO routes with 0.1 ml of the vaccine produced HI antibodies presumed to protective level.

^‡^SPF chickens vaccinated by IN/IO routes with 0.1 ml of the vaccine did not induce detectable HI antibody.
